# Association between micronutrient supplementation during pregnancy and preterm birth: evidence from a large-scale children survey and Mendelian randomization study

**DOI:** 10.3389/fpubh.2025.1451006

**Published:** 2025-05-09

**Authors:** Liwen Ding, Yiliang Liu, Xiaona Yin, Guomin Wen, Dengli Sun, Danxia Xian, Yafen Zhao, Maolin Zhang, Weikang Yang, Weiqing Chen

**Affiliations:** ^1^Department of Epidemiology and Health Statistics, School of Public Health, Sun Yat-sen University, Guangzhou, China; ^2^Department of Neurology, Xiangya Hospital, Central South University, Changsha, China; ^3^Maternal and Child Healthcare Hospital of Longhua District, Shenzhen, China; ^4^School of Health Management, Xinhua College of Guangzhou, Guangzhou, China

**Keywords:** preterm birth, micronutrient supplementation, folic acid, iron, calcium, multinutrient, Mendelian randomization

## Abstract

**Background:**

Preterm birth (PTB) is a leading cause of neonatal mortality and under-five mortality worldwide, with long-term health impacts. While micronutrient supplementation shows promise in preventing PTB, its effectiveness remains controversial due to confounding factors. This study aims to elucidate the association between micronutrient supplementation and PTB risk by analyzing a large-scale children survey and employing Mendelian Randomization (MR) to address confounding factors.

**Methods:**

This study recruited 66,728 mother-child dyads in Longhua District, Shenzhen, China in 2021. Participants provided information on micronutrient supplementation (multinutrient, folic acid, calcium, and iron) through a structured questionnaire. Logistic regression assessed the association between micronutrient supplementation and PTB in crude, adjusted, and full-inclusion models. MR analysis used summary-level GWAS data from the UK Biobank and FinnGen consortiums. The main MR analyses employed inverse variance weighting (IVW), with sensitivity analyses including MR Egger regression, weighted median, weighted mode, simple mode, and MR-PRESSO.

**Results:**

Observational analysis indicated folic acid (OR = 0.80, 95%CI: 0.72–0.89), calcium (OR = 0.88, 95%CI: 0.80–0.96), and iron (OR = 0.92, 95%CI: 0.86–0.98) as protective factors against PTB, especially in co-supplementation, while multinutrient supplementation showed no significant effect. MR analysis indicating a consistent protective effect of calcium (OR_IVW_ = 0.04, 95% CI: 0.004–0.42, *p* < 0.01, *p*_FDR_ <0.05). Sensitivity analyses supported these findings, detecting no bias or pleiotropy.

**Conclusion:**

Combining observational data with genetic causal inference, our study confirms the protective roles of folic acid, calcium, and iron against PTB, with MR particularly highlighting calcium's causal association with reduced PTB risk. These findings provide a comprehensive understanding and underscore the importance of targeted nutritional interventions, especially calcium, in prenatal care for PTB prevention. However, given the limitations of the self-reported data and the lack of information on doses used in our study, future prospective studies with more detailed micronutrient information are needed to provide more comprehensive evidence.

## 1 Introduction

Preterm birth (PTB), defined as infants born before 37 weeks of gestation, is a leading cause of neonatal mortality (1). The World Health Organization reports that in 2020, approximately 9.9% of global births (13.4 million) and 6.1% of births in China (752.9 thousand) were preterm (2). Complications from PTB represent the primary cause of death in children under 5 years old, accounting for nearly 0.94 million deaths worldwide in 2019 and serving as the second leading cause of mortality in this age group in China, with 30,900 deaths in 2015 (3, 4). PTB is also associated with immediate, short-term and long-term physical, neurodevelopmental, and socioeconomic effects, such as growth retardation, psychiatric disorders, and increased risks of chronic diseases, including cardiovascular, respiratory, and endocrine/metabolic disorders in early to mid-adulthood (5–7).

Evidence suggests that most PTBs can be mitigated through cost-effective interventions, particularly those aimed at improving the nutritional status of pregnant women and fetuses (8). Micronutrients, most of which can only be acquired exogenously, support nearly all metabolic activities, thereby facilitating fetal development and maturation into a healthy neonate (9). However, deficiencies in micronutrients like iron, vitamin A, zinc, vitamin B12, and folic acid are prevalent among pregnant women worldwide (10–15). In China, the prevalence of vitamin D deficiency among pregnant women increased from 25.52% in 2012 to 41.96% in 2017 (16). Furthermore, low intake levels of iron, iodine, calcium, and folic acid have also been reported (17–20). Therefore, in the fact of micronutrients remains suboptimal among pregnant women, micronutrient supplementation plays important roles in maintaining nutritional status.

Although there have been studies on the association and mechanisms between micronutrient supplementation and PTB, most of them have been observational, and the results have not always been uniform. A meta-analysis of 91,425 participants from 18 trials showed little significant difference between multiple-micronutrient supplementation and PTB, with the confidence interval just crossing the line of no effect (21). However, a cluster randomized, double-blind trial in Bangladesh demonstrated that multiple-micronutrient supplementation reduced the risk of PTB (22). Despite the fact that prenatal iron and folic acid improved low birthweight, trials did not report significant reduction in PTB (23, 24). The association between vitamin D and PTB also presented inconsistent results across different studies (25–29). These inconsistencies may stem from small sample sizes, uncertain causal time sequences, and confounding biases (9, 26, 30). Consequently, there is a need for well-designed studies with large samples controlling for potential confounders to address these disparities.

Mendelian Randomization (MR) offers a novel approach to establish causality by leveraging genetic variants that are randomly allocated at conception, thereby minimizing confounding and reverse causation in observational studies (31). Previous MR research has focused mostly on adulthood and less on early life. Recent genome-wide association studies (GWAS) on perinatal lifestyle phenotypes and PTB provide an opportunity to investigate these associations without bias. There is a relevant MR study, but only for a single nutrient (32), so the relationship between additional nutrients and PTB remains to be explored by MR.

Our study aims to assess the association between micronutrient supplementation and PTB using a large dataset of mother-child dyads with detailed profiles, and to complement the findings with causal inference via MR analysis.

## 2 Methods

### 2.1 Observational study

#### 2.1.1 Participants

**Figure 1:**
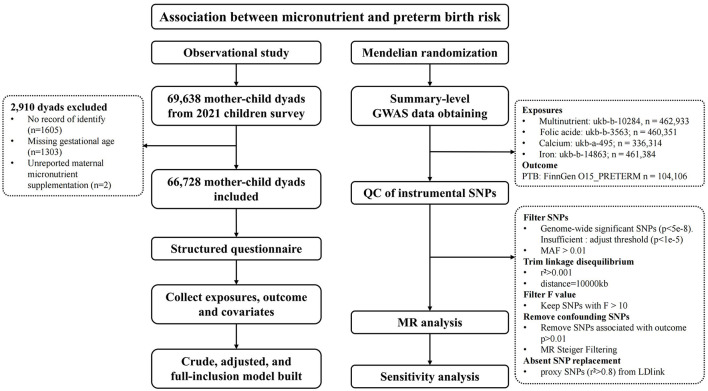
Study profile. GWAS, genome-wide association studies; MAF, minor allele frequency; MR, Mendelian Randomization; QC, Quality Control; SNPs, Single nucleotide polymorphisms.

Participants were recruited from the 2021 children survey, which included 69,638 mother-child dyads from 235 kindergartens in Longhua District, Shenzhen, China. Exclusions were made for cases with no identity record (*N* = 1,605), missing gestational age (*N* = 1,303), and unreported maternal micronutrient supplementation (*N* = 2), resulting in a final sample of 66,728 dyads, see Figure 1. This study received approval from the Ethics Committee of the School of Public Health, Sun Yat-sen University. Informed consent was obtained from all children's primary guardians.

#### 2.1.2 Data acquirement

Data were collected using an online structured questionnaire, which was completed under the supervision of health practitioners and kindergarten teachers. The questionnaire was administered during the children's kindergarten years (typically 3–7 years after birth), and covered demographic details, maternal condition during pregnancy (the medical history, pregnancy complications, behavior and habits, environmental exposure during pregnancy, etc.), and child birth characteristics (gestational age, birth weight, delivery mode, etc.).

Micronutrient supplementation during pregnancy (multinutrient, folic acid, calcium, and iron) was assessed through maternal self-reporting using four separate questions “Did you take multinutrient (supplements containing three or more vitamins with or without minerals)/folic acid/calcium/iron during your pregnancy?” (Answer was “NO” or “YES”). Mothers who reported “YES” were defined as exposure group, respectively, while those who reported “NO” were defined as the reference group. PTB was determined based on the mother's response to the question “What was the gestational age of your child at birth?” A gestational age of <37 weeks was classified as PTB.

Covariates were collected according to studies and the univariate analysis of our research (29, 33–39), including child's information (sex, birth season), maternal characteristic (age of conception, education, marital status, household income, pre-pregnancy BMI, weight gain during pregnancy, parity, and multiple pregnancy), maternal disease (polycystic ovarian syndrome, pregnancy-induced hypertension, pre-eclampsia, gestational diabetes mellitus, and perinatal depression), and behaviors and lifestyle during pregnancy (employment, prenatal care visit). Multiple imputation (MI) was employed for missing covariate data (40), with details and coding provided in Supplementary Table S1.

### 2.2 Mendelian randomization

For each micronutrient supplement as exposure, Summary-level GWAS data for micronutrient supplement were collected from the United Kingdom Biobank (UKB) cohort with European ancestry from MRC-IEU consortium, including multinutrient (Treatment/medication code: multinutrient; dataset ukb-b-10284, *N* = 462,933), folic acid (Vitamin and mineral supplements: Folic acid or Folate; dataset ukb-b-3563, *N* = 460,351), Calcium (Mineral and other dietary supplements: Calcium; dataset ukb-a-495, *N* = 336,314), and iron product (Mineral and other dietary supplements: Iron; dataset ukb-b-14863, *N* = 461,384). For PTB outcome, summary-level GWAS data with sample size of 5,480 cases and 98,626 controls of European descent was obtained from the FinnGen (https://r5.finngen.fi/pheno/O15_PRETERM).

Instrumental variables (IVs) were chosen based on strict criteria to support causal inference, including Single nucleotide polymorphisms (SNPs) selection guidelines to ensure validity and minimize confounding (41). SNPs with a significant threshold (*p* < 1 × 10^−5^) were considered as potential instrumental variables. Besides, each SNP was selected based on the following rules: (1) with a minor allele frequency (MAF) more than 0.01; (2) not possibly being in linkage disequilibrium (*r*^2^ > 0.001, distance = 10,000 kb); (3) adequate strength of IV evaluated by F-statistics (*F* > 10); (4) without an exorbitant association with outcome (*p* > 0.01); (5) avoid reverse causal effect using MR Steiger Filtering. We replaced the SNPs absent in the outcome data with their available corresponding proxy SNPs (*r*^2^ > 0.8) through LDlink (https://ldlink.nci.nih.gov/).

### 2.3 Statistical analysis

In the observational study, associations between micronutrient supplementation during pregnancy and PTB were analyzed using univariate and multivariate logistic regression, under three different models: the crude model, without adjustment for confounders; the adjusted model, adjusted for selected confounders; and the full-inclusion model, included all micronutrients in the model and adjusted for all confounders to address the confounding effects of co-supplementation.

In the MR process, the effect size estimates the association of the SNP with the specific phenotype expressed as β (i.e., ln OR). The Wald ratio was obtained by dividing the β-outcome by the β-exposure. Since there are more than one SNP was available, the inverse variance weighting (IVW) was considered an optimum approach for meta-analysis after vindicating the absence of horizontal pleiotropy and heterogeneity (42). For multiple comparison, the false discovery rate (FDR) correction was utilized to adjust the *p*-values. Sensitivity analysis includes MR Egger regression (43), weighted median (44), weighted mode (45), simple mode, and MR-PRESSO (46). MR Egger intercepts and Cochran Q statistics were conducted to evaluate heterogeneity and pleiotropy.

Statistical analyses above were performed via R version 4.2.3, with the “TwoSampleMR” v0.5.6 and “MR PRESSO” v1.0 packages. *P*-value of <0.05 was deemed statistically significant.

## 3 Results

### 3.1 Evidence from observational study

Table 1 presents the characteristics of the children survey sample. The mean age of mothers at conception was 29.12 years, with a mean pre-pregnancy BMI was 20.75 Kg/m^2^. Among the 66,728 mothers analyzed, 85.1% had attained at least a high school education, 97.4% were married, 38.3% gained <10 kg during pregnancy, 45.0% were multiparous, approximately half of the households earned <RMB 20,000 every month, 93.5% had prenatal care visit and 66.2% were employed during pregnancy. Pregnancy complications varied, with perinatal depression at 0.3% and gestational diabetes mellitus at 7.4%. Over half of the children were male, 2.5% were from multiple pregnancies, and births were evenly distributed across seasons. Micronutrient supplementation rates were 41.3% for multinutrient, 92.8% for folic acid, 86.9% for calcium, and 40.1% for iron. The prevalence of PTB was 8.0%.

**Table 1:** Characteristics of the study participants from 2021 children survey (*N* = 66,728).

**Variables**	**Description**
	**Mean** ±***SD***
Maternal age of conception (years)	29.12 ± 4.40
Pre-pregnancy BMI (Kg/m^2^)	20.75 ± 2.83
	***N*** **(%)**
Maternal education (high school and greater than high school)	55,607 (85.1)
Marital status (married)	65,013 (97.4)
Household income (<RMB 20,000)	33,239 (49.8)
Household income (RMB 20,000–39,999)	22,295 (33.4)
Maternal weight gain ( ≤ 10 kg)	25,544 (38.3)
Parity (multiparous)	29,952 (45.0)
Multiple pregnancy (yes)	1,698 (2.5)
Polycystic ovarian syndrome (yes)	1,842 (2.8)
Pregnancy-induced hypertension (yes)	1,297 (2.0)
Pre-eclampsia (yes)	210 (0.3)
Gestational diabetes mellitus (yes)	4,878 (7.4)
Perinatal depression (yes)	175 (0.3)
Employment (yes)	44,174 (66.2)
Prenatal care visit (yes)	55,643 (93.5)
Child's sex (male)	35,573 (53.3)
Birth season (spring)	15,615 (23.4)
Birth season (summer)	16,259 (24.4)
Birth season (autumn)	18,280 (27.4)
Multinutrient (yes)	27,585 (41.3)
Folic acid (yes)	61,897 (92.8)
Calcium (yes)	57,999 (86.9)
Iron (yes)	26,762 (40.1)
Preterm birth (yes)	5,328 (8.0)

The results of covariates selection are available in Supplementary Table S2. In the crude model, all four micronutrients were protective factors for PTB. In the adjusted model, compared to the reference group, supplementation with multinutrient (OR = 0.91, 95%CI: 0.85–0.96), folic acid (OR = 0.73, 95%CI: 0.66–0.80), calcium (OR = 0.79, 95%CI: 0.73–0.86) and iron (OR = 0.88, 95%CI: 0.82–0.93) were all inversely associated with PTB (Table 2).

**Table 2:** Associations between micronutrient supplementation during pregnancy and preterm birth in crude and adjusted model (*N* = 66,728).

**Variables**	**Preterm birth**		
	***N*** **(%)**	**Crude**	**Adjusted** ^a^
		***OR*** **(95%*****CI*****)**	***OR*** **(95%*****CI*****)**
**Multinutrient**			
No	3,210 (8.2)	1.00	1.00
Yes	2,118 (7.7)	0.93 (0.88, 0.99)^*^	0.91 (0.85, 0.96)^**^
**Folic acid**			
No	548 (11.3)	1.00	1.00
Yes	4,780 (7.7)	0.65 (0.60, 0.72)^***^	0.73 (0.66, 0.80)^***^
**Calcium**			
No	873 (10.0)	1.00	1.00
Yes	4,455 (7.7)	0.75 (0.69, 0.81)^***^	0.79 (0.73, 0.86)^***^
**Iron**			
No	3,388 (8.5)	1.00	1.00
Yes	1,940 (7.2)	0.84 (0.80, 0.89)^***^	0.88 (0.82, 0.93)^***^

^*^p <0.05, ^**^p <0.01, ^***^p <0.001.

^a^We adjusted for child's information, maternal characteristic, maternal disease, and behaviors and lifestyle during pregnancy.

**Figure 2:**
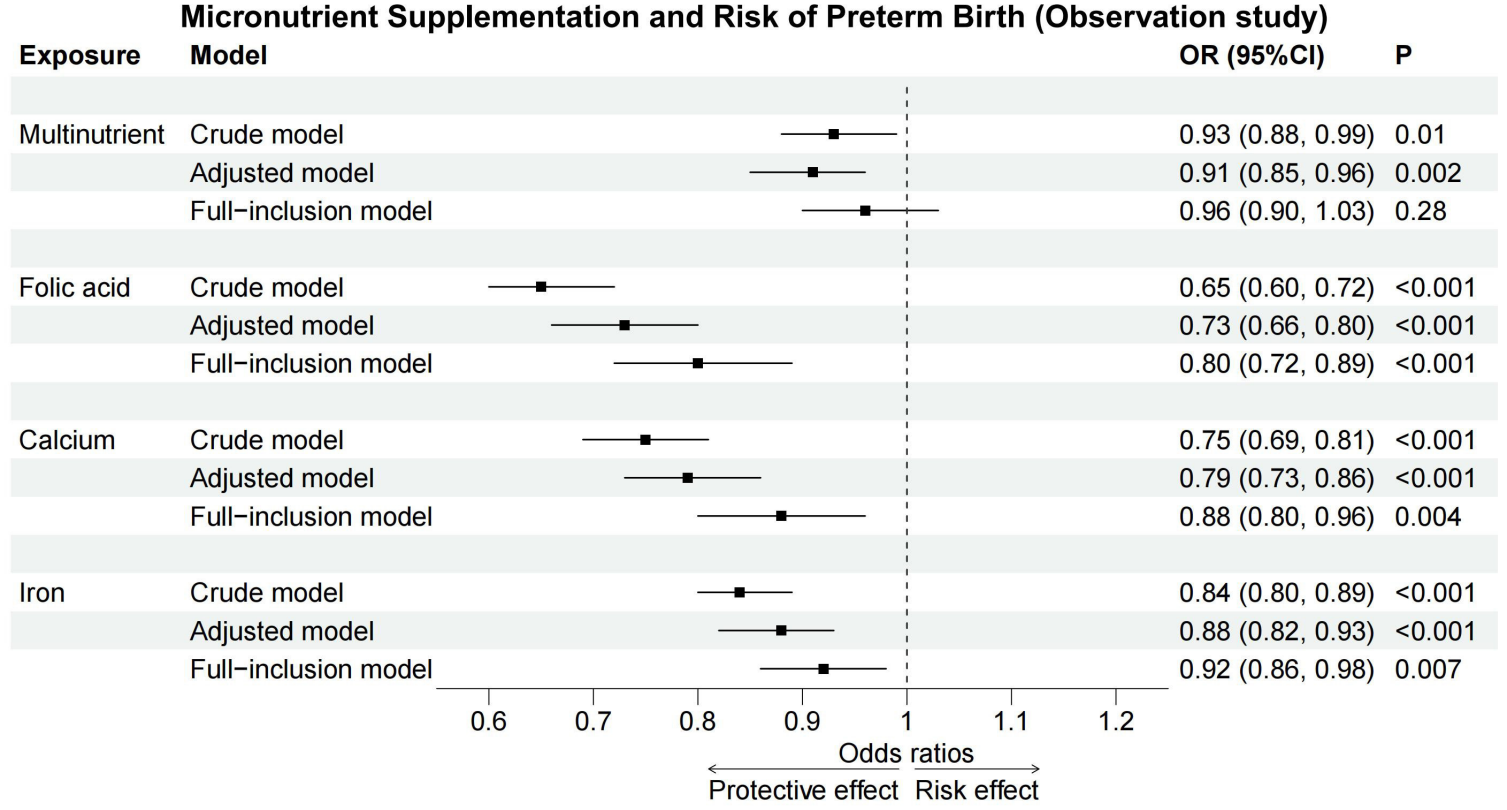
Forest plot of observation study. Across crude, adjusted, and full-inclusion model, folic acid, calcium, and iron acted as protective factors against PTB, while multinutrient supplementation showed no significant effect in full-inclusion model. CI, confidence interval; OR, odds ratio.

Multivariate logistic regression analyses, controlling for other micronutrients, confirmed folic acid (OR = 0.80, 95%CI: 0.72–0.89), calcium (OR = 0.88, 95%CI: 0.80–0.96), and iron (OR = 0.92, 95%CI: 0.86–0.98) as protective against PTB. However, the protective effects were reduced, and multinutrient showed no significant effect (Table 3).These associations across crude, adjusted, and full-inclusion model analyses were illustrated in Figure 2.

**Table 3:** Associations between micronutrient supplementation during pregnancy and preterm birth in full-inclusion model (*N* = 66,728)^a^.

**Variables**	***OR* (95%*CI*)**
Multinutrient (yes)^b^	0.96 (0.90, 1.03)
Folic acid (yes)^b^	0.80 (0.72, 0.89)^***^
Calcium (yes)^b^	0.88 (0.80, 0.96)^**^
Iron (yes)^b^	0.92 (0.86, 0.98)^**^

^**^p <0.01, ^***^p <0.001.

^a^We put all micronutrient supplementation, child's information, maternal characteristic, maternal disease, and behaviors and lifestyle during pregnancy in a model.

^b^Answer “No” as reference.

To further explore the variation in the protective effect of multinutrient, the co-supplementation characteristics of different micronutrients are presented in Supplementary Table S3. A stratified analysis of the association between multinutrient supplementation and PTB under the other three micronutrient supplementation scenarios revealed a marginal statistical difference between the two folic acid subgroups, suggesting a potential interaction between multinutrient and folic acid (Supplementary Table S4).

### 3.2 Mendelian randomization and sensitivity analyses

MR analyses revealed a significant causal relationship between calcium intake and reduced PTB risk, with OR_IVW_ of 0.04 (95%CI: 0.004–0.42, *p* < 0.01, Table 4). After the FDR correction, calcium still significantly associated with lower risk of PTB (*p*_FDR_ <0.05). For other micronutrients, results were less conclusive. Folic acid and iron supplementation displayed considerable uncertainty in their associations with PTB (OR_IVW_ = 13.14, 95% CI: 0.02–8,436.32 for folic acid; OR_IVW_ = 2.13, 95% CI: 0.01–248.88 for iron), and multinutrients also showed no significant correlation, albeit with marginal significance (OR_IVW_ = 0.01, 95% CI: 0.001–1.26, *p* = 0.061). Detailed information about the genetic instrumental variables used for MR analysis is presented in Supplementary Table S5.

**Table 4:** Associations between micronutrient supplementation during pregnancy and preterm birth using IVW methods in MR analysis (*N* = 66,728).

**Variables**	***OR* (95%*CI*)**
Multinutrient (yes)^a^	0.01 (0.001, 1.26)
Folic acid (yes)^a^	13.14 (0.02, 8,436.32)
Calcium (yes)^a^	0.04 (0.004, 0.42)^**^
Iron (yes)^a^	2.13 (0.01, 248.88)

^**^p <0.01.

^a^Answer “No” as reference.

**Figure 3:**
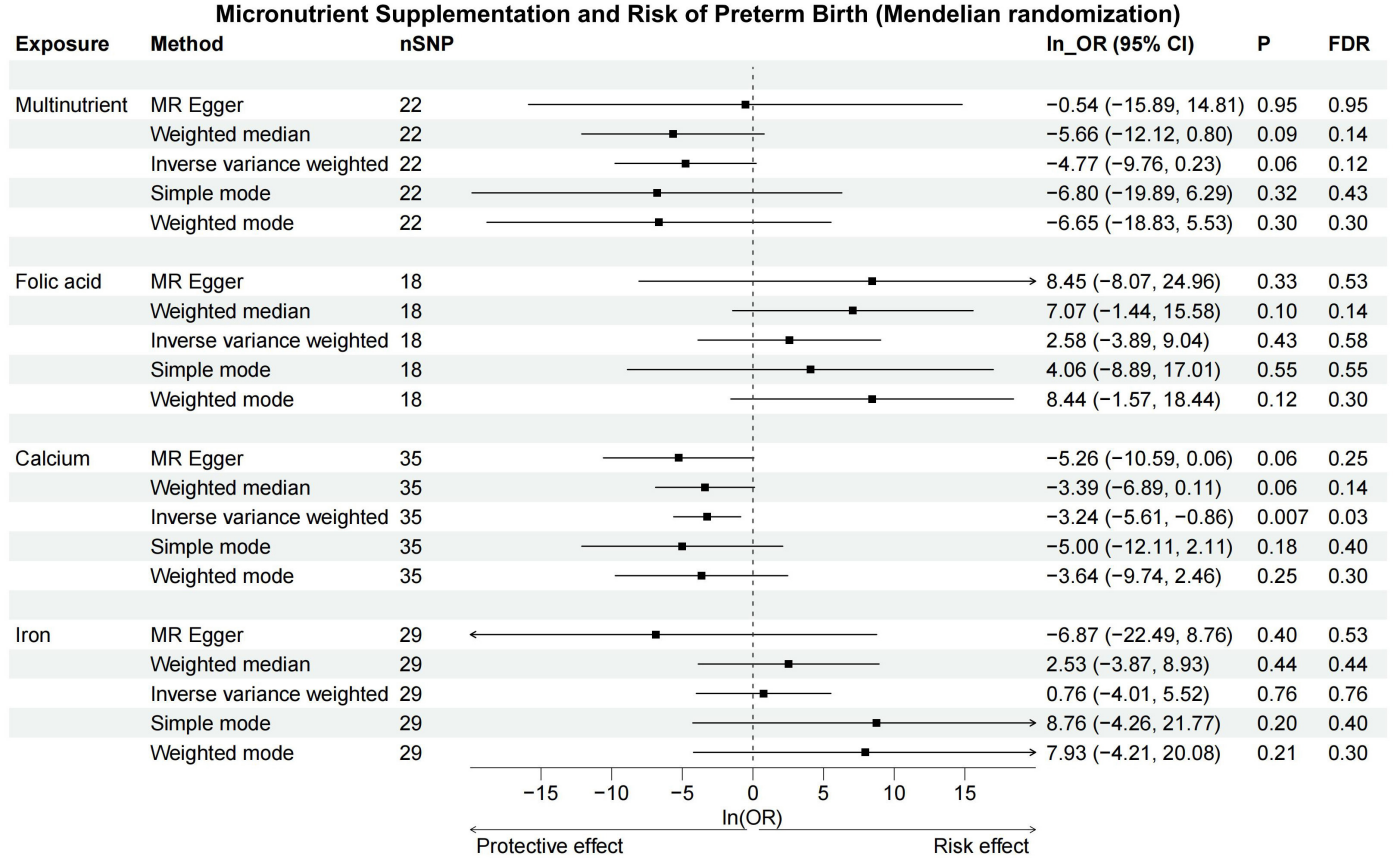
Forest plot of Mendelian randomization. The IVW and weighted median methods indicate a significant reduction in PTB risk with increased calcium intake, but no significant associations were found for multinutrient, folic acid, or iron supplements. CI, confidence interval; FDR, false discovery rate; OR, odds ratio; SNP, single nucleotide polymorphisms.

Sensitivity analysis results, highly consistent with the IVW method, are documented in Supplementary Table S6 and illustrated in Figure 3 and Supplementary Figure S1, showcasing a uniform trend across various MR methods. Forest plots and leave-one-out analyses for each SNP indicated no significant bias from outlier SNPs in the association between micronutrient supplementation and PTB (Supplementary Figures S2, S3). Funnel plots and the MR Egger intercept test found no evidence of directional pleiotropy or significant heterogeneity across all micronutrient supplementation analyses (Supplementary Figure S4). According to the Q-test, there is no conspicuous evidence supporting heterogeneity in the results of all micronutrient supplementations. There is also no suggestion of pleiotropy detected by the MR Egger intercept test among exposures. No horizontal pleiotropy was addressed in either the preliminary study or validation of the MR-PRESSO global test (Supplementary Tables S7–S9).

### 3.3 Integrated observational and MR findings

**Figure 4:**
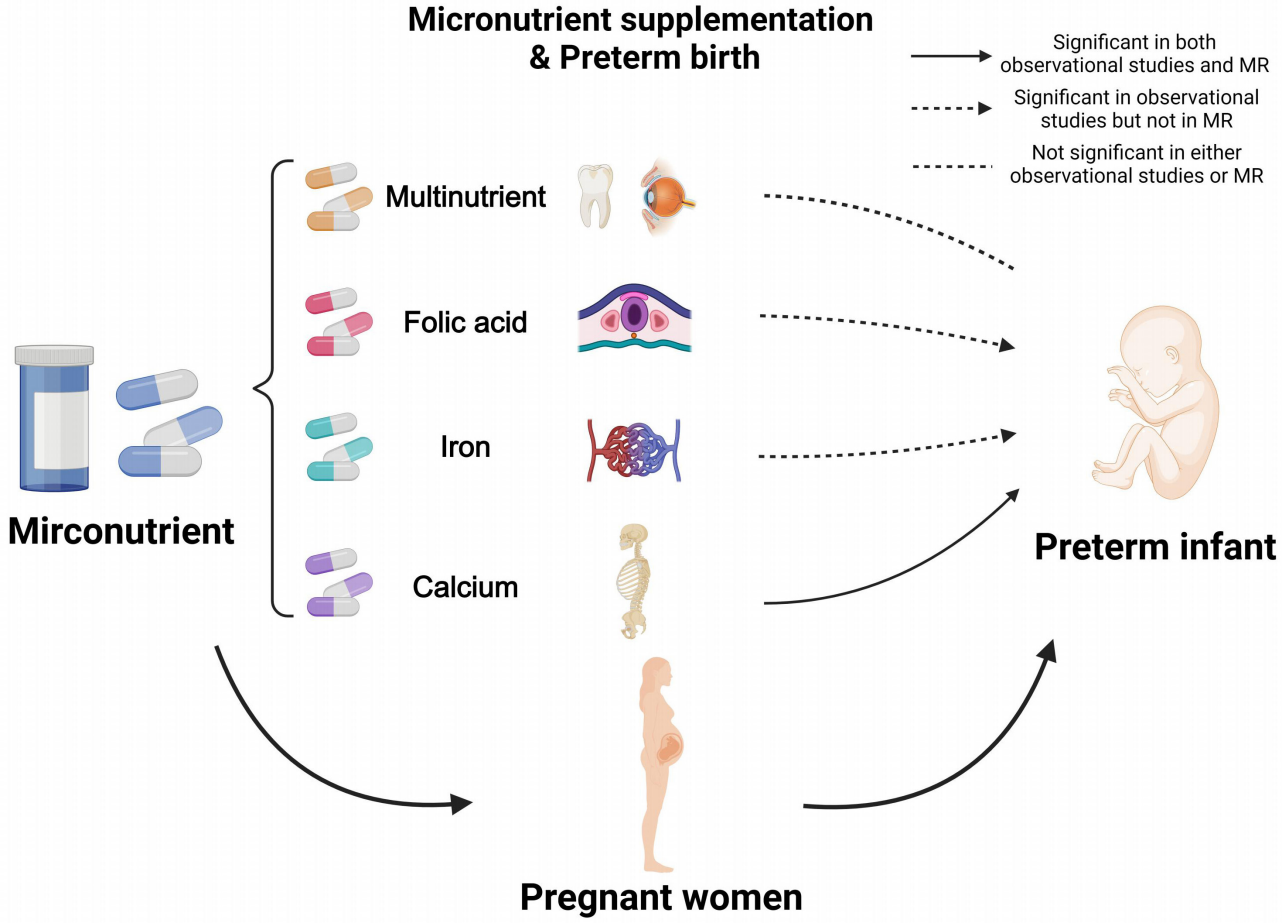
Results of combining observational study and Mendelian randomization. Calcium supplementation showed a significant protective effect against PTB in both analyses, whereas folic acid and iron were significant only in observational analyses, and multinutrient were not significant in either. MR, Mendelian Randomization. Created in BioRender. Ding, R. (2025) https://BioRender.com/itsfbzv.

Combining observational and MR findings, calcium supplementation demonstrated a significant protective effect against PTB in both analytical approaches. In contrast, folic acid and iron were significant only in observational analyses, while multinutrient supplementation showed no significant association in either analytical approach, as illustrated in Figure 4.

## 4 Discussion

In our study based on the 2021 children survey, folic acid, calcium, and iron supplementation were associated with a reduced risk of PTB, particularly in co-supplementation. MR analysis further supported a potential causal relationship between calcium supplementation and lower PTB risk, as indicated by maternal genetic instruments. No significant association was observed between multinutrient supplementation and PTB in either observational or MR analysis.

Similar to our findings, studies have found that not only did multinutrient supplementation fail to significantly reduce the risk of PTB (47–49), but supplementation with individual vitamins, such as vitamins A, B12, and D, also showed no significant association (27–29, 50–52). One possible explanation for these non-significant findings is the heterogeneity among studies and the low doses of vitamins used (53). Interestingly, when measuring the levels of these vitamins in the body, such as 25OHD levels, an association with PTB is often observed (33, 51, 54–56). This discrepancy may stem from the fact that the associations between oral vitamin supplementation and PTB are influenced by confounding factors, whereas vitamin levels measured in the blood are less affected by environmental variables (57, 58). Notably, while crude and adjusted models in our study showed an association between multinutrient supplementation and PTB, this association disappeared after controlling for other micronutrients. This observation aligns with other studies, suggesting that interactions between multinutrient and other micronutrients may drive the observed significant association rather than indicating an independent effect of multinutrient (59). Further high-quality research is needed to better understand these complex interactions in the future.

Folic acid, a water-soluble vitamin B that cannot be synthesized by the body, is significant in neural tube development and is the only perinatal supplement recommended to prevent fetal neural tube defects (60, 61). Observational studies showed that folic acid supplementation reduces the risk of PTB (62), while randomized controlled trials reported no statistically significant effects (63), as did our study. Iron is critical for the delivery of blood oxygen to the fetus, and nearly 40% of pregnant women worldwide suffer from anemia, primarily due to iron deficiency (64, 65). Several meta-analyses included randomized controlled trials showed little or no association between iron supplementation and PTB, as the CI for the pooled effect for PTB just crossed the line of no effect (23, 24). However, other researchers have found that either iron deficiency or excess in pregnant women can lead to PTB, presenting a U-shaped curve for risk associated with iron supplementation (66–68). These results indicate that more prospective controlled trials, as well as more detailed dosage data, are still needed before any firm conclusions can be drawn for these micronutrients.

Calcium is involved in the formation of fetal bones and teeth, as well as in physiological processes such as nerve conduction and muscle contraction. Calcium supplementation may reduce hypertension as well as excessive uterine muscle contractions, as calcium is known to suppress the renin-angiotensin system and decrease the contraction of vascular smooth muscle cells (69–71). Results similar to our results were also seen in cross-sectional and prospective cohort studies (72, 73). Notably, after controlling for other micronutrients, the protective effect of calcium was reduced, suggesting a potential synergistic effect of co-supplementation. However, studies have revealed that simultaneous supplementation of vitamin D and calcium may increase the risk of PTB (53, 59). These contradictory findings warrant careful consideration for pregnant women receiving both vitamin D and calcium supplementation.

MR employs genetic variants as IVs to deduce causal relationships from observational datasets, contingent upon certain prerequisites. These prerequisites include: (1) a robust association of IVs (SNPs) with the targeted exposures; (2) IVs' independence from confounders; and (3) IVs influencing the outcome solely via the exposures (74). MR has the potential to surmount typical observational study challenges, including confounding, reverse causation, and measurement errors (75). Nonetheless, MR findings necessitate prudent interpretation due to possible impacts from heterogeneity, pleiotropy, and GWAS quality. Further research conducted two-sample MR incorporating multiple sensitivity analyses to mitigate these biases (76).

Previous MR study suggested a lack of evidence for a causal relationship between Vitamin D deficiency and PTB, as indicated by an OR (95% CI) of 1.01 (0.93–1.10) (32). This finding is partially corroborated by our MR results concerning multinutrient use. Our MR study further confirmed that calcium intake had a significant causal effect on decreased risk of PTB, as indicated by the negative and significant OR_IVW_. The results remained stable across various sensitivity analyses, indicating no heterogeneity or pleiotropy. Therefore, in addition to folic acid and iron, which are recommended by the WHO's essential drug list for pregnant women (77, 78), our study also suggests that calcium supplementation may be a potential customized preventive approach for PTB.

There are potential limitations to this study. Firstly, the 2021 children survey utilized a structured questionnaire to collect data retrospectively, which may have introduced recall bias. Micronutrient supplementation was defined using binary self-reported questions by mothers, without collecting detailed information on the doses of specific micronutrients, potentially missing certain dose-response relationships, and information on the components of multinutrient and their interactions. Additionally, the types of micronutrients collected in the survey were limited, which constrained the comprehensiveness of the findings. Future research should include more detailed information such as dosage, specific components, and a wider variety of micronutrients, to provide more comprehensive evidence. Second, the observational study predominantly involved an Asian population, whereas the MR analysis was based on a European population. This potential discrepancy in the population demographics may influence the generalizability of the causal inferences drawn. Third, despite adhering to stringent selection criteria for instrumental variables to confirm the genetic instruments' quality, the current dearth of comprehensive GWAS on PTB limits our findings. Further studies, therefore, should aim for independent validation utilizing GWAS on larger prospective PTB studies to strengthen causal interpretations.

## 5 Conclusion

In conclusion, our findings indicate that folic acid, calcium, and iron are protective factors against PTB, with MR analysis supporting calcium's causal association with reduced PTB risk. Neither observational nor MR analyses showed a significant effect of multinutrient supplementation on PTB. These results provide a comprehensive understanding of the association between micronutrient supplementation during pregnancy and PTB, and underscore the importance of calcium supplementation in preventing PTB, thereby informing more comprehensive guidelines for pregnant women. Future prospective studies with more detailed information on micronutrients are crucial to draw definitive conclusions about micronutrient prevention of PTB.

## Data Availability

The raw data supporting the conclusions of this article will be made available by the authors, without undue reservation.
