# Supplementary material for: Association between micronutrient supplementation during pregnancy and preterm birth: evidence from a large-scale children survey and Mendelian randomization study
Source: Front Public Health. 2025 May 9;13:1451006. doi: 10.3389/fpubh.2025.1451006 (PMC12101085; doi:10.3389/fpubh.2025.1451006)
Supplement: Supplementary file 1 [file Data_Sheet_1.zip › S_Table_1_question&code.docx]

**Supplemental Table 1 Definitions and coding used in the study.**

| Variable | Question | Values |
| --- | --- | --- |
| Multinutrient supplementation | Did you take multinutrient (supplements containing three or more vitamins with or without minerals) during your pregnancy? | 1= No  2= Yes |
| Folic acid supplementation | Did you take folic acid (supplements containing folic acid only) during your pregnancy? | 1= No  2= Yes |
| Calcium supplementation | Did you take calcium (supplements containing calcium only) during your pregnancy? | 1= No  2= Yes |
| Iron supplementation | Did you take iron (supplements containing iron only) during your pregnancy? | 1= No  2= Yes |
| Preterm birth | What was the gestational age of child (week)? | 1= No (≥37 weeks)  2= Yes (<37 weeks) |
| Maternal age of conception | What is the mother's birth date?  What is the child's birth date? | Current mother's age - current child's age |
| Pre-pregnancy BMI | What was the mother's height (m) and weight (kg) before this pregnancy? | Weight (kg) ÷ height ^2 (m) |
| Maternal education | What is the mother's education? | 1= Less than high school  2= High school and greater than high school |
| Marital status | What is the mother's current marital status? | 1= Not married (Single, divorced and widowed)  2= Married (Married and remarried) |
| Household income | What is your total monthly household income? | 1= <RMB 20,000  2= RMB 20,000–39,999  3= >RMB 39,999 |
| Maternal weight gain | What was the total weight gain of the mother during the pregnancy with the child? | 1= ≤10 kg  2= >10 kg |
| Parity | How many times did the mother give birth before this child? | 1= Nulliparous  2= Multiparous |
| Multiple pregnancy | How many fetuses was the mother carrying? | 1 = Single pregnancy  2 = Multiple pregnancy |
| Polycystic ovarian syndrome | Was the mother ever diagnosed with Polycystic Ovarian Syndrome (POS) before she was pregnant with this child? | 1= No  2= Yes |
| Pregnancy-induced hypertension | Was the mother diagnosed with pregnancy-induced hypertension during pregnancy with this child? | 1= No  2= Yes |
| Pre-eclampsia | Was the mother diagnosed with pre-eclampsia during pregnancy with this child? | 1= No  2= Yes |
| Gestational diabetes mellitus | Was the mother diagnosed with gestational diabetes mellitus during pregnancy with this child? | 1= No  2= Yes |
| Perinatal depression | Was the mother diagnosed with perinatal depression during pregnancy with this child? | 1= No  2= Yes |
| Employment | Was the mother employed during pregnancy with this child? | 1= No  2= Yes |
| Prenatal care visit | Did the mother have prenatal care visits during pregnancy with this child? | 1= No  2= Yes |
| Child’s sex | What is the sex of the child? | 1= Male  2= Female |
| Birth season | What is the child's birth date? | 1= Spring  2= Summer  3= Autumn  4= Winter |
