# Supplementary material for: Association between micronutrient supplementation during pregnancy and preterm birth: evidence from a large-scale children survey and Mendelian randomization study
Source: Front Public Health. 2025 May 9;13:1451006. doi: 10.3389/fpubh.2025.1451006 (PMC12101085; doi:10.3389/fpubh.2025.1451006)
Supplement: Supplementary file 1 [file Data_Sheet_1.zip › S_Table_2_Univariate analysis.docx]

**Supplemental Table 2 Univariate analysis of the association between participants’ characteristics and PTB for the covariates selection.**

| Variables | Preterm birth | | |
| --- | --- | --- | --- |
|  | *N* (%) | *OR*(95%*CI*) | *P* |
| Maternal age of conception | - | 1.03 (1.02,1.04) | <0.001 |
| Pre-pregnancy BMI | - | 1.03 (1.02,1.04) | <0.001 |
| Maternal education |  |  |  |
| Less than high school | 892 (9.2) | 1.00 |  |
| High school and greater than high school | 4,291 (7.7) | 0.83 (0.77,0.90) | <0.001 |
| Marital status |  |  |  |
| Not married | 192 (11.2) | 1.00 |  |
| Married | 5,136 (7.9) | 0.68 (0.58,0.79) | <0.001 |
| Household income |  |  |  |
| <RMB 20,000 | 2,801 (8.4) | 1.00 |  |
| RMB 20,000–39,999 | 1,659 (7.4) | 0.87 (0.82,0.93) | <0.001 |
| >RMB 39,999 | 867 (7.7) | 0.91 (0.84,0.99) | 0.02 |
| Maternal weight gain |  |  |  |
| ≤10 kg | 2,452 (9.6) | 1.00 |  |
| >10 kg | 2,875 (7) | 0.71 (0.67,0.75) | <0.001 |
| Parity |  |  |  |
| Nulliparous | 2,735 (7.5) | 1.00 |  |
| Multiparous | 2,578 (8.6) | 1.17 (1.10,1.23) | <0.001 |
| Multiple pregnancy |  |  |  |
| No | 4,649 (7.1) | 1.00 |  |
| Yes | 678 (39.9) | 8.63 (7.80,9.56) | <0.001 |
| Polycystic ovarian syndrome |  |  |  |
| No | 4,966 (7.8) | 1.00 |  |
| Yes | 226 (12.3) | 1.66 (1.44,1.91) | <0.001 |
| Pregnancy-induced hypertension |  |  |  |
| No | 4,944 (7.6) | 1.00 |  |
| Yes | 315 (24.3) | 3.89 (3.41,4.43) | <0.001 |
| Pre-eclampsia |  |  |  |
| No | 5,179 (7.8) | 1.00 |  |
| Yes | 91 (43.3) | 9.00 (6.84,11.84) | <0.001 |
| Gestational diabetes mellitus |  |  |  |
| No | 4,792 (7.8) | 1.00 |  |
| Yes | 496 (10.2) | 1.34 (1.21,1.47) | <0.001 |
| Perinatal depression |  |  |  |
| No | 5,227 (7.9) | 1.00 |  |
| Yes | 23 (13.1) | 1.75 (1.13,2.72) | 0.01 |
| Employment |  |  |  |
| No | 1,950 (8.6) | 1.00 |  |
| Yes | 3,378 (7.6) | 0.88 (0.82,0.93) | <0.001 |
| Prenatal care visit |  |  |  |
| No | 340 (8.8) | 1.00 |  |
| Yes | 4,382 (7.9) | 0.89 (0.79,1.00) | 0.04 |
| Child’s sex |  |  |  |
| Male | 2,971 (8.4) | 1.00 |  |
| Female | 2,357 (7.6) | 0.90 (0.85,0.95) | <0.001 |
| Birth season |  |  |  |
| Spring | 1,225 (7.8) | 1.00 |  |
| Summer | 1,305 (8) | 1.02 (0.94,1.11) | 0.55 |
| Autumn | 1,394 (7.6) | 0.97 (0.90,1.05) | 0.45 |
| Winter | 1,404 (8.5) | 1.09 (1.00,1.18) | 0.04 |
