# Supplementary material for: Association between micronutrient supplementation during pregnancy and preterm birth: evidence from a large-scale children survey and Mendelian randomization study
Source: Front Public Health. 2025 May 9;13:1451006. doi: 10.3389/fpubh.2025.1451006 (PMC12101085; doi:10.3389/fpubh.2025.1451006)
Supplement: Supplementary file 2 [file Data_Sheet_2.pdf]

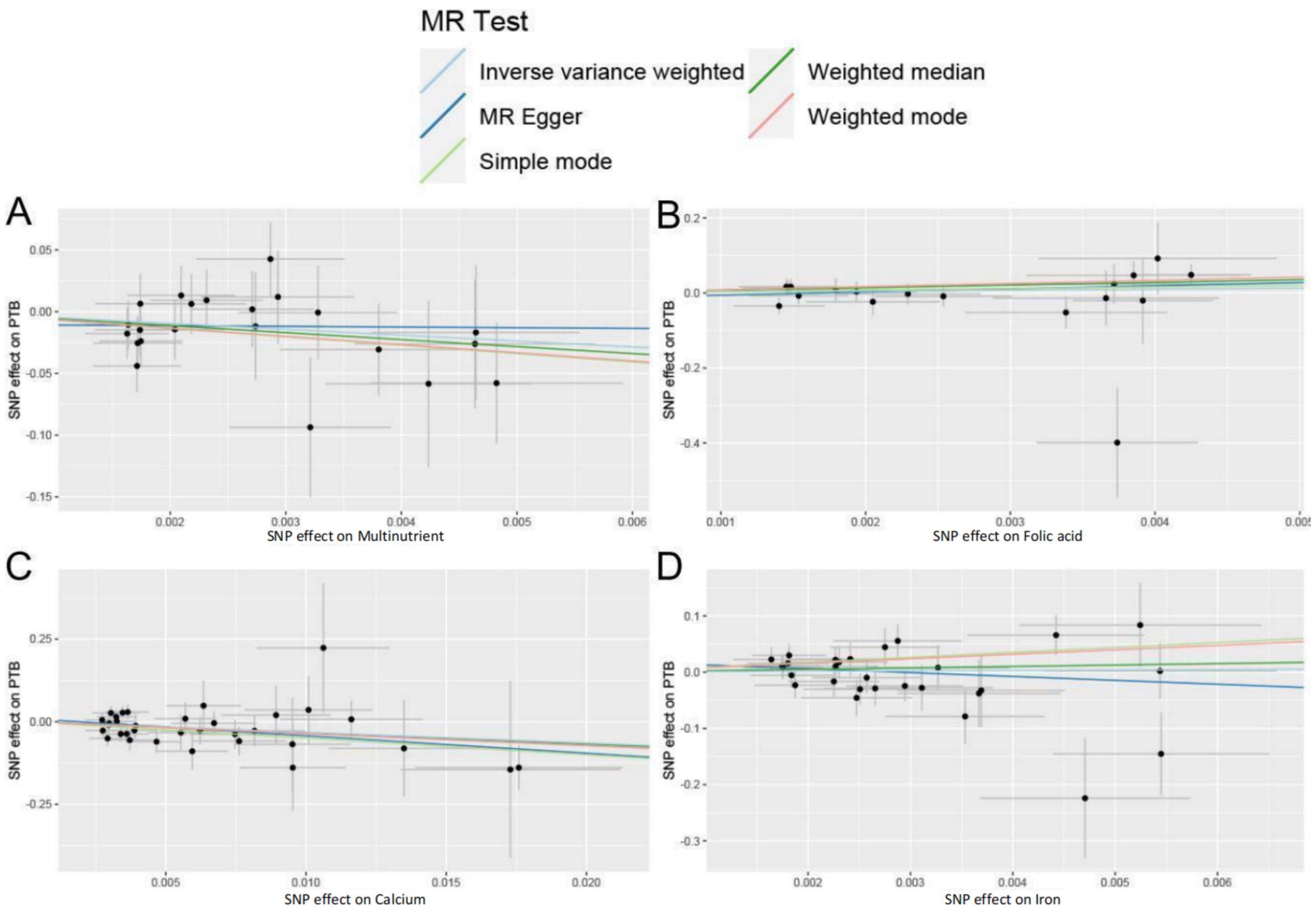

Supplemental Fig 1. Sensitivity of Five Methods in Mendelian Randomization

The results of sensitivity analyses showed a high consistency with inverse variance weighting (IVW) method in multinutrient (A), folic acid (B), calcium (C) and iron (D) intake and preterm birth (PTB).

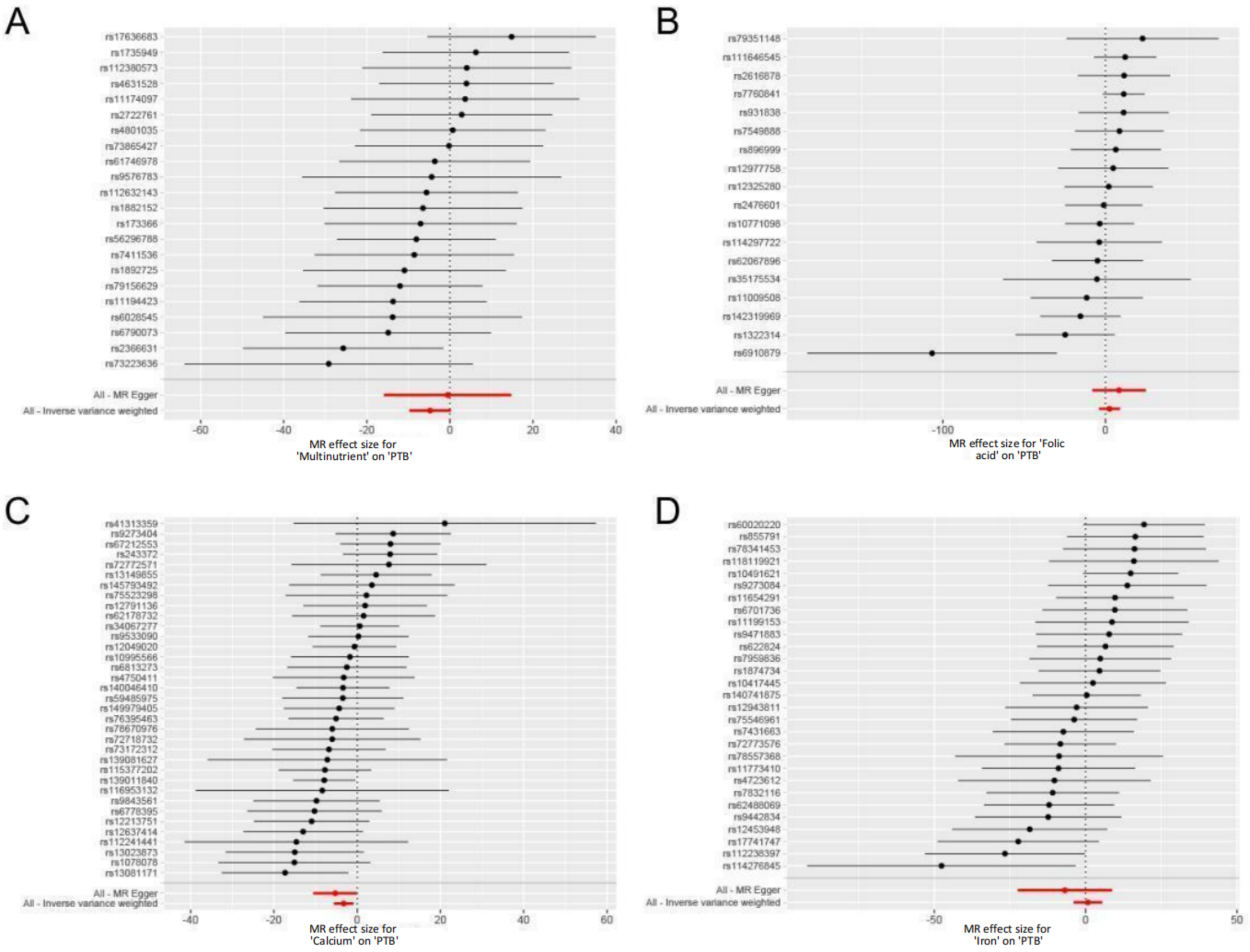

Supplemental Fig 2. Forest Plots of the SNPs

The forest plots of each SNP presented no obvious bias in Mendelian randomization of multinutrient (A), folic acid (B) ,calcium (C) and iron (D) intake and preterm birth (PTB).

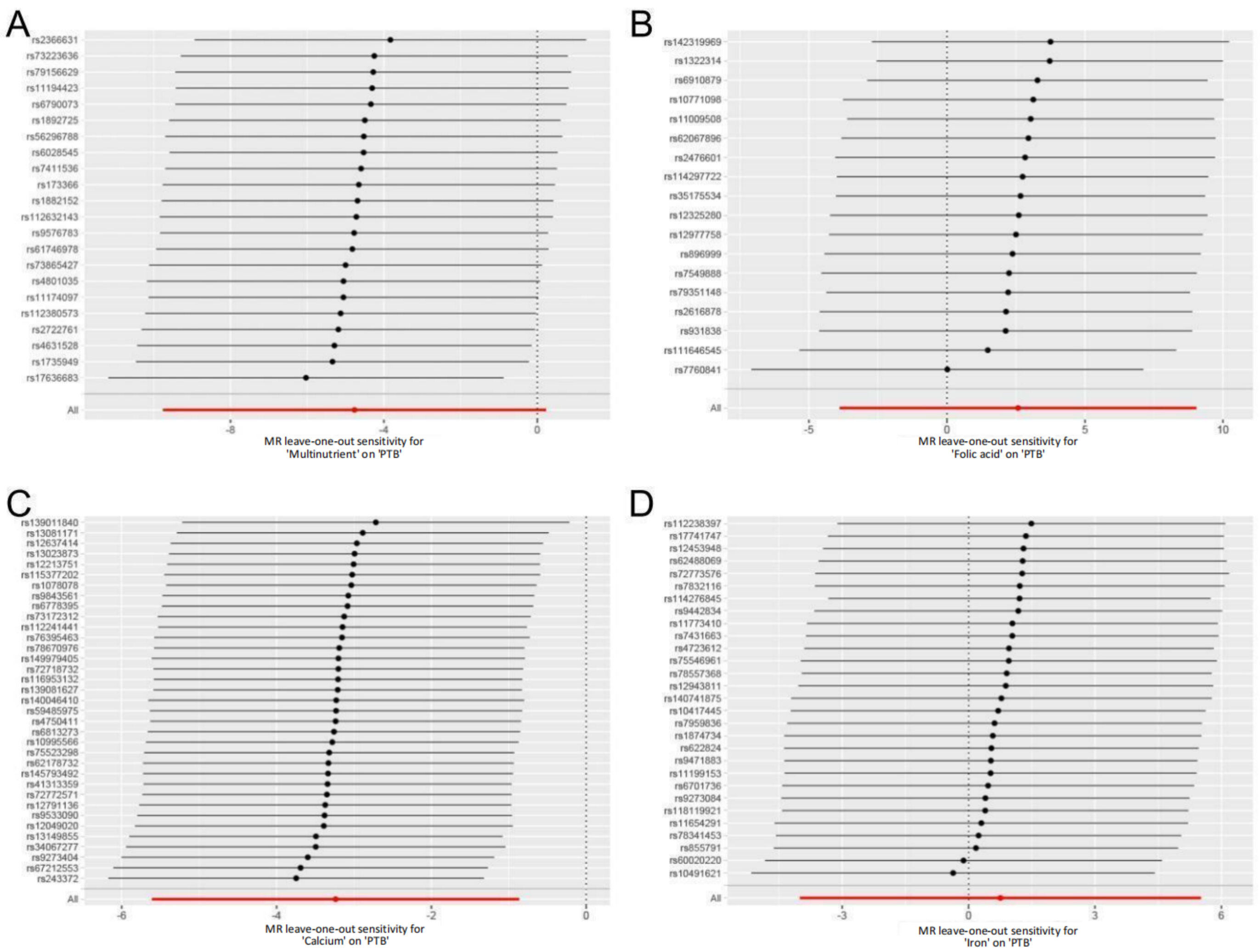

Supplemental Fig 3. Forest Plots of the Leave-one-out Analyses

The forest plots of the leave-one-out analyses of SNPs presented no outlier instrumental variables in Mendelian randomization analyses of multinutrient (A), folic acid (B), calcium (C) and iron (D) intake and preterm birth (PTB).

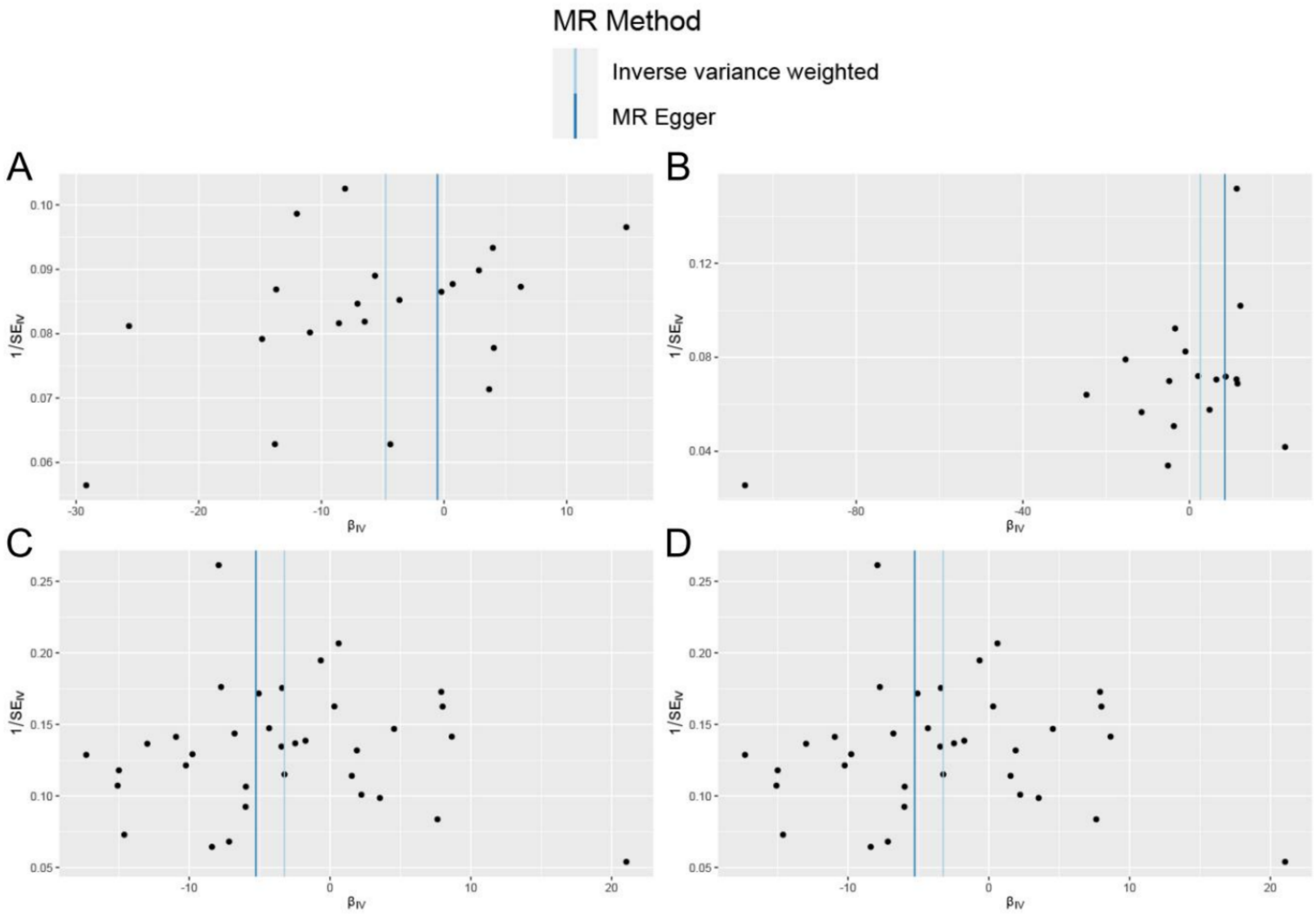

Supplemental Fig 4. Funnel Plots of the Mendelian Randomization

Funnel plots showed that compared to MR Egger methods, there is no evidence of directional pleiotropy in IVW methods in multinutrient (A), folic acid (B), calcium (C) and iron (D) intake and preterm birth (PTB).
